# Under conditions closely mimicking vaginal fluid, *Lactobacillus jensenii* strain 62B produces a bacteriocin-like inhibitory substance that targets and eliminates *Gardnerella* species

**DOI:** 10.1099/mic.0.001409

**Published:** 2023-11-01

**Authors:** Stephany Navarro, Habib Abla, Jane A. Colmer-Hamood, Gary Ventolini, Abdul N. Hamood

**Affiliations:** ^1^​ Department of Immunology and Molecular Microbiology, Texas Tech University Health Sciences Center, Lubbock, TX, USA; ^2^​ School of Medicine, Texas Tech University Health Sciences Center, Lubbock, TX, USA; ^3^​ Department of Medical Education, Texas Tech University Health Sciences Center, Lubbock, TX, USA; ^4^​ Department of Obstetrics and Gynecology, Texas Tech University Health Sciences Center Permian Basin, Odessa, TX, USA; ^5^​ Department of Surgery, Texas Tech University Health Sciences Center, Lubbock, TX, USA

**Keywords:** antibacterial activity, bacteriocin, bacteriocin-like inhibitory substance, cell-free supernatant, *Gardnerella*spp, *Lactobacillus jensenii*, medium simulating vaginal fluid

## Abstract

Within the vaginal ecosystem, lactobacilli and *Gardnerella* spp. likely interact and influence each other’s growth, yet the details of this interaction are not clearly defined. Using medium simulating vaginal fluid and a two-chamber co-culturing system to prevent cell-to-cell contact between the bacteria, we examined the possibility that *Lactobacillus jensenii* 62B (Lj 62B) and/or *G. piotii* (Gp) JCP8151B produce extracellular factors through which they influence each other’s viability. By 24 h post-inoculation (hpi) in the co-culture system and under conditions similar to the vaginal environment – pH 5.0, 37 °C, and 5% CO_2_, Lj 62B viability was not affected but Gp JCP8151B had been eliminated. Cell-free supernatant harvested from Lj 62B cultures (Lj-CFS) at 20 hpi, but not 16 hpi, also eliminated Gp JCP8151B growth. Neither lactic acid nor H_2_O_2_ production by Lj 62B was responsible for this effect. The Lj-CFS did not affect viability of three species of lactobacilli or eight species of Gram-positive and Gram-negative uropathogens but eliminated viability of eight different strains of *Gardnerella* spp. Activity of the inhibitory factor within Lj-CFS was abolished by protease treatment and reduced by heat treatment suggesting it is most likely a bacteriocin-like protein; fractionation revealed that the factor has a molecular weight within the 10–30 kDa range. These results suggest that, in medium mimicking vaginal fluid and growth conditions similar to the vaginal environment, Lj 62B produces a potential bacteriocin-like inhibitory substance (Lj-BLIS) that clearly targets *Gardnerella* spp. strains. Once fully characterized, Lj-BLIS may be a potential treatment for *Gardnerella-*related BV that does not alter the vaginal microflora.

## Introduction

The microflora of the vaginal ecosystem of a healthy premenopausal woman are maintained in equilibrium through interactions among the various microorganisms [1, 2]. Lactobacilli, which contribute to a healthy system, are prevalent microorganisms among the vaginal microflora [1, 3]. At least 70 % of the total bacteria identified within the reproductive system of healthy pre-menopausal women are lactobacilli [2, 4]. Based on several reports, the most prevalent *Lactobacillus* spp. within the vagina of a healthy woman are *L. crispatus, L. jensenii*, *L. gasseri *, and *L. iners* [2, 5, 6]. However, the dominance of each species varied among different ethnic groups. For example, White and Asian women have higher prevalence of *L. crispatus* and *L. jensenii* as compared to Black and Hispanic women [2]. In a woman of reproductive age, the high level of oestrogen results in the deposition of increased amounts of glycogen within the vaginal epithelium, which is metabolized by the vaginal lactobacilli to produce organic acids, specifically lactic acid [7, 8]. As a result, the physiological pH of the vagina is moderately acidic, ranging from 3.1 to 4.1 (mean 3.5), which inhibits the growth of certain vaginal pathogens [9, 10]. Some vaginal lactobacilli such as *L. crispatus* and *L. jensenii* also produce hydrogen peroxide (H_2_O_2_), which is bactericidal for certain vaginal pathogens [11, 12]. Furthermore, the vaginal lactobacilli produce antimicrobial peptides/proteins (bacteriocins) to eliminate competitive bacteria and protect the ecological niche of the bacteria that produce them [13, 14]. Bacteriocins differ from antibiotics by being ribosomally synthesized [15]. Although bacteriocins vary in their mode of action, many of them permeabilize the cell causing the efflux of amino acids and ions which deplete the pH gradient, while others degrade the cell wall [16–18]. However, bacteriocins do not cause vaginal irritation and are not toxic to vaginal epithelial cells *in vitro* [19, 20].

Bacterial vaginosis (BV), the most common vaginal infection in females aged 15–44 years, results from the disruption of the normal vaginal microflora [21, 22]. *Gardnerella vaginalis* is often detected in low numbers among the vaginal microflora of healthy women [23]. During BV, the prevalent lactobacilli are replaced with a higher concentration of strict and facultative anaerobic bacteria including *Prevotella *, *Mobiluncus *, *Ureaplasma *, *Mycoplasma *, and *Gardnerella* [24–26]. Recently, *G. vaginalis* was resolved into multiple species constituting four clades; four of these species – *G. vaginalis* (clade 1)*, G. piotii* (clade 2)*,* and *G. swidsinskii* and *G. leopolidii* (clade 4) – have been associated with BV [27–29]. Hill and Albert found that *G. vaginalis, G. piotii,* and *G. swidsinskii* were associated with vaginal symptoms of BV by whole genome sequencing [27], while others found *G. leopoldii* and *G. swindsinskii* to be associated with a healthy vaginal microbiome [30]. Prominent features associated with BV are an increase in the vaginal pH (above 4.5), a decrease in antimicrobial activity of the vaginal fluid in infected women, and impairment of many of the innate immune pathways (compared with healthy women) [31, 32]. Although BV is not a life-threatening disease, it predisposes women to sexually transmitted diseases such as human immunodeficiency virus, pelvic inflammatory disease, and preterm birth, endometriosis, and infertility [33–36]. *Gardnerella* spp. produce several virulence factors including pili, microcapsules, vaginolysin (vaginal hemolysin), phospholipase C, proteases, siderophores, and sialidases [37, 38]. Besides these, *Gardnerella* spp. carry genes that code for cytoadhesions, exopolysaccharide for biofilm formation, and antimicrobial resistance systems, as well as genes that may enhance their ability to compete with and exclude other vaginal bacteria [39]. The three most important factors for BV are sialidase and vaginal hemolysin production and biofilm formation [30]. While all four of the *Gardnerella* spp. associated with BV produce vaginal hemolysin and form biofilms, members of clades 1–3 (*G. vaginalis, G. piotii,* and *Gardnerella* spp.), but not clade 4 (* G. leopoldii* and *G. swindsinskii*), produce sialidase [30]. Treatment of BV with antibiotics such as clindamycin and metronidazole is standard and can be effective, but the recurrence rate is high with one study reporting 3.6 % recurrence at 1 month and 28 % by 6 months [40] and up to 30–40 % reported by others [41, 42]. The resistance to metronidazole is also associated with specific *Gardnerella* spp., with species in clades 3 and 4 exhibiting 100 % resistance, clade 1 (* G. vaginalis *) 35 % resistance, and clade 2 (* G. piotii *) only 7.1 % resistance [30]. Besides the risk of the emergence of antibiotic resistant pathogens associated with the use of intravaginal antimicrobial products, it has recently been shown that antibiotic treatment disturbs the healthy vaginal microflora [41, 43]. As an alternative, the administration of probiotic species of lactobacilli, which are not major constituents of the vaginal flora such as *Lacticaseibacillus* (*Lcb*.) *rhamnosus, L. acidophilus, Lcb. casei, Lactiplantibacillus plantarum,* and *Ligilactobacillus salivarius *, have been utilized with varying degrees of success [40, 44, 45]. The probiotic bacteria do not remain within the vaginal microbiota when dosing ceases, thus reducing the long-term benefit [45]. While probiotic lactobacilli are considered safe, there have been increasing reports of serious infections caused by several of these strains, especially *Lcb. rhamnosus* and *L. acidophilus *, over the past 3 years, especially in immunocompromised patients [46, 47]. Therefore, utilizing antimicrobial products generated by lactobacilli, rather than the lactobacilli themseles, is a promising alternative for treating *Gardnerella-*related vaginal infections and as a product that could be applied prophylactically in women prone to BV recurrence.

Previous studies suggested that within the vaginal microbiota of a healthy woman, lactobacilli produce defensive factors that limit the growth of potential pathogens including *Gardnerella* spp. [1, 48, 49]. In turn, *Gardnerella* spp. respond with their own countermeasures to help them persist or colonize the vaginal mucosa [37–39]. However, most of these findings are based on the characterization of the lactobacilli or *Gardnerella* spp. in laboratory media designed to enhance their growth and production of their virulence factors. To understand the potential interplay between the lactobacilli and *Gardnerella* spp. *in vivo*, it is important to utilize an *in vitro* medium that closely mimics the environment within the vagina and supports the growth of both lactobacilli and *Gardnerella* spp. Such a medium, the medium simulating vaginal fluid (MSVF) [50], supported the viability of *G. piotii* (Gp) JCP8151B and the vaginal lactobacilli *L. jensenii* 62B (Lj 62B), *L. gasseri* 63 AM, and *L. crispatus* JV-V01 individually under different pH conditions for an extended period (30 d) [51]. In this study, we used the Transwell two-chamber co-culture technique, which prevents direct contact between the bacteria in the upper and lower chambers, to determine if extracellular products synthesized by these vaginal *Lactobacillus* spp. (*L. crispatus, L. gasseri*, and *L. jensenii *) would inhibit Gp JCP8151B or vice versa. Gp JCP8151B did not affect the growth of any of the lactobacilli after 5 days of co-culture; but while *L. crispatus* and *L. gasseri* reduced Gp JCP8151B growth, Lj 62B inhibited its growth completely. Characterization of the inhibitory factor showed it is likely a bacteriocin-like inhibitory substance (Lj-BLIS) with a narrow spectrum of activity, affecting only *Gardnerella* spp. and making the Lj-BLIS a possible treatment for BV.

## Methods

### Bacterial strains, media, and growth conditions

Strains used in this study are listed in Table 1. *Lactobacillus* and *Gardnerella* strains were obtained from the American Type Culture Collection (Manassas, VA, USA) or the BEI Resources Repository (Manassas, VA, USA). At the time of acquisition, all *Gardnerella* strains were identified as *G. vaginalis* by the repositor; however, the published emended descriptions [29] and resolution of species [27] have indicated that our strains include three *G. vaginalis *, three *G. piotii *, one *G. leopoldii *, and one *Gardnerella* gsp12 (Table 1). In fact, the *Gardnerella* test strain JCP8151B used in the majority of described experiments is now considered to be *G. piotii* (Table 1) [27–29, 52]. Urinary isolates (four species of Gram-positive cocci, four species of Gram-negative bacilli, and one yeast species) were obtained from patients with urinary tract infections seen at the Texas Tech University Health Sciences Centre Urology Clinic under an IRB approved protocol and deidentified prior to use in this study (Table 1). Frozen stock cultures of Lj 62B, *L. gasseri* 63 AM (Lg 63AM), *L. crispatus* JV-V01 (Lc JV-V01), Gp JCP8151B, and other *Gardnerella* spp. were grown at 37 °C under 5 % CO_2_ for 48 h in de Man, Rogosa, and Sharpe broth (MRSB) (Oxoid Limited, Basingstoke, Hampshire, UK) or New York City broth (NYCB), respectively, as recommended by suppliers of the strains (ATCC and BEI). Bacterial urinary isolates were grown from frozen stock cultures in tryptic soy broth (RPI, Mount Prospect, IL, USA) and the yeast was grown in yeast peptone dextrose broth (BD Difco, Franklin Lakes, NJ, USA) at 37 °C for 24 h. MRSB, tryptic soy broth, and yeast peptone dextrose broth were prepared according to the manufacturers’ directions; NYCB was made according to the ATCC Medium 1685 formulation (https://www.atcc.org/). Medium simulating vaginal fluid (MSVF) was used for all experiments conducted in this study; its composition and preparation are described in Table 2 [50]. Following 24 h or 48 h of growth in laboratory media, 1 ml aliquots of the cultures were pelleted and resuspended in MSVF three times to remove residual media. After the final resuspension, the cells were used to adjust fresh MSVF to an OD_600_ 0.02 (or less as appropriate depending on microorganism) to yield an initial inoculum of 10^4^ colony forming units (c.f.u.) per millilitre. Lj 62B was used in all experiments as the producer of the inhibitory substance and Gp JCP8151B served as the target strain. All experiments examining the spectrum of activity of the inhibitory substance and its nature were done in MSVF pH 5.0 incubated for specified times at 37 °C under 5 % CO_2_.

**Table 1.** Strains used in this study

Strain	Origin of isolate	Source	Reference(s)
* Gardnerella vaginalis* 317	Woman with BV	ATCC 14019	[27, 29, 52]
* Gardnerella vaginalis* JCP7275	Woman with BV	BEI HM-1105	[27, 29, 52]
* Gardnerella vaginalis* JCP7276	Woman with BV	BEI HM-1106	[27, 29, 52]
* Gardnerella* (*vaginalis*) *piotii* JCP8066	Healthy woman	BEI HM-1112	[27, 29, 52]
* Gardnerella* (*vaginalis*) *piotii* JCP8070	Woman with BV	BEI HM-1113	[27, 29, 52]
*** Gardnerella* (*vaginalis*) *piotii* JCP8151B**	Woman with BV	BEI HM-1116	[27, 29, 52]
*Gardnerella (vaginalis) leopoldii* AMD	Woman with BV	BEI NR-50514	[27, 29, 52]
* Gardnerella* (*vaginalis*) gsp12 CMW7778B	Pregnant woman	BEI HMI-1298	[27, 52]
* Lactobacillus crispatus* JV-V01	Human vaginal flora	BEI HM-103	[51]
* Lactobacillus gasseri* 63 AM	Human	ATCC 33323	[51]
*** Lactobacillus jensenii* 62B**	Vaginal discharge	ATCC 25258	[51]
*Candida albicans* UI-017-Ca	Patient with UTI	Clinical isolate	[73]
* Enterobacter cloacae* UI-095-Enc	Patient with UTI	Clinical isolate	[73]
* Enterococcus faecalis* UI-031-Efc	Patient with UTI	Clinical isolate	[73]
* Escherichia coli* UI-001-Ec	Patient with UTI	Clinical isolate	[73]
* Klebsiella pneumoniae* UI-002-Kp	Patient with UTI	Clinical isolate	[73]
* Pseudomonas aeruginosa* UI-040-Pa	Patient with UTI	Clinical isolate	[73]
*Staphylococcus aureus,* methicillin resistant UI-009-MRSA	Patient with UTI	Clinical isolate	[73]
* Staphylococcus epidermidis* UI-086-Se	Patient with UTI	Clinical isolate	[73]
* Streptococcus agalactiae* UI-056-Sag	Patient with UTI	Clinical isolate	[73]

Bold font indicates producer strain and test target strain used in majority of experiments.

ATCC, American Type Culture collection; BEI, BEI Resources; BV, bacterial vaginosis; UTI, urinary tract infection.

**Table 2.** Composition and preparation of MSFV

Compound	Final concn (g l^−1^)	Preparation	Sterilization	Assembly of medium
Glucose	10.00	Dissolve together in 0.89 L dH_2_O (pH 4.2–4.3)	Autoclave*	890 ml
Lactic acid	2.00			
Acetic acid	1.00			
NaCl	3.50			
KCl	1.50			
Tween 80	1.064			
Glycogen	10.00	20 % solution†	Autoclave	50 ml
Albumin	2.00	5 % solution	Filter (0.22 µm membrane)	40 ml
Mucin	0.25	1.33 % solution	Autoclave	18.8 ml
Urea	0.50	40 % solution	Autoclave	1.25 ml
Total volume				1000 ml
Final pH				4.25±0.05

*Autoclaving was done at 121 °C with 15 psi for 15 min.

†All solutions were made in dH_2_O pH 4.2–4.3.

### Two chamber co-culture experiments

Co-culture experiments were conducted using two-chambered Transwell plates (Corning, Glendale, AZ, USA) to physically separate *Gardnerella* spp. from the lactobacilli while allowing the medium and factors produced by the bacteria to flow across a permeable membrane. Both chambers of the Transwell plate were filled with 750 µl of MSVF. The upper chamber was inoculated with 10^4^ c.f.u. of Gp JCP8151B and the lower chamber with 10^4^ c.f.u. of Lj 62B, Lg 63 AM, or Lc JV-V01. For controls, only one chamber was inoculated with the tested strain. Plates were sealed with a gas permeable membrane (Breathe easy, RPI) to prevent desiccation and incubated for up to 5 d post-inoculation (dpi). After the designated time, tenfold serial dilutions were performed, 10 µl aliquots of the dilutions of the tested *Lactobacillus* strain or *Gardnerella* cultures were spotted on MRS agar or chocolate agar plates, respectively, and incubated at 37 °C under 5 % CO_2_ for 48 h to quantify the amount (c.f.u. ml^−1^) of bacteria present.

### Collection of Lj 62B cell-free supernatant (Lj-CFS) for use as a growth medium

Lj 62B was grown in MSVF in 24-well microtitre plates (Costar, Corning, Durham, NC, USA) for 16, 20, 22, or 24 h. Supernatants from the Lj 62B cultures were harvested by centrifugation at each time point, pooled, and filtered (0.45 µm membrane syringe filter; Whatman, Cytiva, Marlborough, MA, USA) to remove any residual bacteria. The cell-free supernatant (Lj-CFS) was then used as a growth medium. Gp JCP8151B or Lj 62B (10^4^ c.f.u. ml^−1^) were inoculated in 1 ml of Lj-CFS from each time point in a 24-well microtitre plate. After 24 h, Gp JCP8151B or Lj 62B growth was quantitated. Additional experiments to examine the spectrum of activity of Lj-CFS against seven other *Gardnerella* strains, *L. gasseri* and *L. crispatus *, and the nine urinary isolates were conducted using Lj-CFS from the 24 h time point only (Lj-CFS24). To determine the time required for the inhibitory substance present in Lj-CFS to act on Gp JCP8151B, Lj-CFS24 was inoculated with 10^4^ c.f.u. ml^−1^ of the strain, incubated at 37 °C under 5 % CO_2_, and samples were collected and the c.f.u. per millilitre of Gp JCP8151B present was determined at 0.5, 1, 2, and 4 h post-inoculation (hpi). Filtered MSVF (fMSVF) was used as a control in all experiments using Lj-CFS.

### Transmission electron microscopy (TEM)

Gp JCP8151B was inoculated into fMSVF or Lj-CFS24 at 10^5^ c.f.u. ml^−1^ and incubated for 1 h, the time at which growth of Gp JCP8151B was reduced by one log, at 37 °C under 5 % CO_2_. Bacteria from the control and treated cultures were pelleted, washed in PBS, and fixed with 2.5 % glutaraldehyde for 24 h at 4 °C. Fixed cells were washed three times using 0.05 M cacodylate buffer to remove glutaraldehyde, post-fixed using 1 % osmium tetroxide for 1 h, and washed again with 0.05 M cacodylate buffer. Samples were then dehydrated using graded ethanol concentrations (25–100 %) and embedded in EPON 828 liquid epoxy resin (Miller Stephenson Chemical Company, Kagel Canyon, CA). Embedded samples were ultra-thin sectioned (85 nm), coated on a copper grid, and stained with uranyl acetate and lead citrate. The grids were examined using a Hitachi H-7650 transmission electron microscope (Hitachi, Tokyo, Japan).

### Quantitation of l- and d-lactic acid and H_2_O_2_

To determine the amount of l- and/or d-lactic acid or H_2_O_2_ produced by Gp JCP8151B and Lj 62B, strains were grown individually for 24 h in MSVF, the cells were pelleted, and the supernatants were collected. The R-Biopharm E Lactic Acid Test Kit (Darmstadt, Germany) was used to determine the levels of l- and d-lactic acid and the Pierce Quantitative Peroxide Assay Kit (Thermo Scientific, Rockford, IL, USA) was used to assess the amount of H_2_O_2_ produced within the supernatants. Each kit was used according to the manufacturer’s directions. To evaluate the effect of these compounds on Gp JCP8151B growth, d-lactic acid or H_2_O_2_ (Acros Organics, Waltham, MA, USA) or both were exogenously added to MSVF at 1× or 2× the level produced by Lj 62B. The MSVF was inoculated with 10^4^ c.f.u. ml^−1^ of Gp JCP8151B, incubated for 24 h, and the c.f.u. per millilitre were determined.

### Heat and protease treatment of the Lj inhibitory substance

Lj-CFS24 contains a substance that is inhibitory for *Gardnerella* spp. To determine if the inhibitory substance was sensitive to heat denaturation, Lj-CFS24 and filtered MSVF (fMSVF) (as a control) were boiled (100 °C) for 15 or 30 min or autoclaved (121 °C at 15 psi) for 15 min. Each heat-treated sample was cooled to room temperature and then mixed 50 % (v/v) with untreated fMSVF to replace nutrients likely degraded by heat treatment. Gp JCP8151B was inoculated into each mixture at 10^4^ c.f.u. ml^−1^ in 24-well microtitre plates and incubated as described above. Samples were obtained at 24 hpi and the c.f.u. per millilitre were determined.

To examine the protease sensitivity of the Lj inhibitory substance, Lj-CFS24 and fMSVF were treated with trypsin (Thermo Scientific) or pepsin (MilliporeSigma, St. Louis, MO, USA) at a concentration of 1 mg ml^−1^ and incubated for 2 h at 37 °C. Protease-treated samples were mixed 50 % (v/v) with untreated fMSVF prior to use. The treated mixtures were inoculated with 10^4^ c.f.u. ml^−1^ of Gp JCP8151B, incubated as described above, and the c.f.u. per millilitre determined 24 hpi.

### Molecular weight range of the Lj inhibitory substance

Lj-CFS24 and fMSVF were fractionated using 100-, 30-, 10-, and 5 kDa molecular weight cut off (MWCO) columns according to the manufacturer’s instructions (Vivaspin, Cytiva). Filtrates from each fractionation column were mixed 50 % (v/v) with untreated fMSVF prior to use. Gp JCP8151B was inoculated into each mixture at 10^4^ c.f.u. ml^−1^ in 24-well microtitre plates, incubated for 24 h, and the c.f.u. per millilitre was determined.

### Determining if Lj-BLIS is produced upon the growth of Lj 62B in MRSB

We grew Lj 62B in MRSB pH 6.2 ± 0.2 at 37 °C under 5 % CO_2_ for 24 h, harvested the supernatant, and filtered it to remove any bacteria and cell debris (Lj-MRSB-CSF24). The Lj-MRSB-CSF24 was then fractionated using a 30 kDa MWCO column as above. To remove the inhibitory levels of H_2_O_2_ and d-lactic acid present when Lj 62B is grown in MRSB, the filtrate containing proteins <30 kDa was subsequently fractionated using a 10 kDa MWCO column. The retentate containing the 10–30 kDa proteins of the Lj-MRSB-CSF24 was collected and resuspended in NYCB to yield a 6× concentrated fraction (fLj-MRSB-CFS24). As a control, we used the same approach to fractionate MRSB (fMRSB). The prepared filtrates were mixed 1 : 6 (v/v) with NYCB, inoculated with Gp JCP8151B at 10^4^ c.f.u. ml^−1^ in 24-well microtitre plates. The plates were incubated for 24 h at 37 °C under 5 % CO_2_ and the c.f.u. per millilitre was determined.

### Statistical analyses

GraphPad Prism version 9.4.0 (673) (GraphPad Software, San Diego, CA, USA) was used for all statistical analyses. Data represent the means±SEM of three independent experiments for each group (*n*=3). The c.f.u. data were routinely log transformed prior to graphing and statistical analysis. One-way ANOVA with Dunnett’s multiple comparisons posttest was used to compare each treatment to fMSVF or appropriate control; one-way ANOVA with Tukey’s multiple comparisons posttest was used to compare differences among the fractions (all pairs of pairs) or with Šídák’s multiple comparisons posttest to compare selected pairs. Two-tailed unpaired *t* tests were used to compare individual pairs. Statistical significance is shown as *, *P*<0.05; **, *P*<0.01; ***, *P*<0.001; ****, *P*<0.0001; ns, no significant difference.

## Results

### Lj 62B eliminated Gp JCP8151B as early as 20 hpi without direct contact

**Fig. 1.**
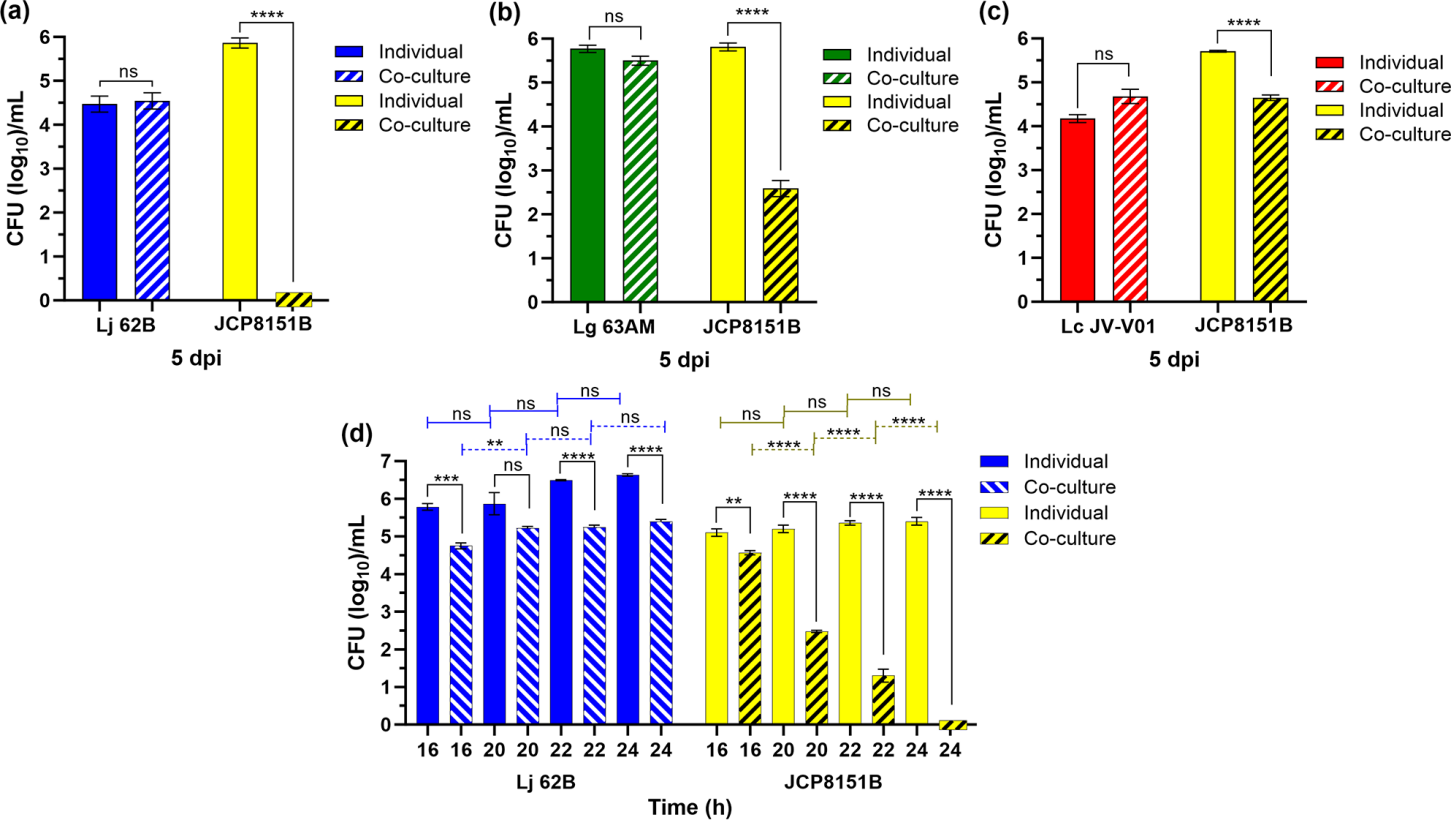
*
L. jensenii* 62B (**a**), *L. gasseri* 63 AM (**b**), and *L. crispatus* JV-V01 (**c**) affect the growth of Gp JCP8151B at five dpi during co-culture. (**d**) The effect of Lj 62B on Gp JCP8151B growth begins at 20 hpi with eradication by 24 hpi. Bars represent the means of three independent experiments±SEM. Significant differences between individual and co-cultures for each microorganism were determined by unpaired two-tailed *t*-test (**a**), (**b**), and (**c**). Significant changes in c.f.u. over time were determined by one way ANOVA with Šídák’s multiple comparison posttest comparing selected pairs as indicated on graph (**d**); black brackets, control (individual culture) to co-culture at each time point; solid blue or dark yellow brackets, individual cultures compared from time point to time point; dashed blue or dark yellow brackets, co-cultures compared from time point to time point. ns, no significant difference; *, *P*<0.05; **, *P*<0.01; ***, *P*<0.001; ****, *P*<0.0001.

Within the vaginal microbiome, these species may interact through direct contact or via products secreted by one or more of the species. To explore the effect that the vaginal *Lactobacillus* spp. may have on the growth of *Gardnerella* spp., or vice versa, we first analysed the effect of potential extracellular factors using Transwell plates which allow diffusion of extracellular factors released by the bacteria to cross a transmembrane between the top and bottom chambers but prevent bacterial cell-to-cell contact. At five dpi, there were no significant differences between the c.f.u. of individual cultures of Lj 62B, *L. gasseri *, or *L. crispatus* and their c.f.u. in co-culture with Gp JCP8151B; however, the c.f.u. of Gp JCP8151B were significantly reduced when co-cultured with any of the three *Lactobacillus* spp. (Fig. 1a–c). Since the greatest reduction in c.f.u. occurred in the Gp JCP8151B-Lj 62B co-culture (Fig. 1a), and since we had previously found that the growth of Lj 62B was more robust in MSVF at pH 5.0 [51], we continued our analyses using Lj 62B. To investigate this phenomenon further, and to determine if this effect might occur at an earlier time point during the co-culturing, we repeated the experiment but obtained samples at 16, 20, 22, and 24 hpi. While the c.f.u. per millilitre of Lj 62B co-cultured with Gp JCP8151B increased gradually over the time points, the c.f.u. of Gp JCP8151B, which were similar to those of Lj 62B at 16 hpi (~5 logs), were significantly reduced by two logs at 20 hpi and three logs at 22 hpi with no c.f.u. of Gp JCP8151B recovered at 24 hpi (Fig. 1d). This suggests that Lj 62B secreted a soluble product that affected Gp JCP8151B growth at 20 hpi that was present in sufficient quantity to completely eradicate it by 24 hpi. It is possible that rather than eliminating Gp JCP8151B growth, the Lj-CFS24 reduced it below our detection limits. In our established protocol, we assessed bacterial growth at different time points by determining the c.f.u. within 10 µl aliquots of the 1 ml cultures, or 1/100 vol of the cultures. To address this possibility, we enhanced our detection limits by assessing the c.f.u. within 100 -µl aliquots of the culture or 1/10 vol. However, even within the 100 -µl aliquots, we detected no c.f.u. in the Lj-CFS24 treated cultures (data not shown).

### Lj 62B cell-free supernatant obtained at 20 hpi, but not 16 hpi, eliminated Gp JCP8151B

**Fig. 2.**
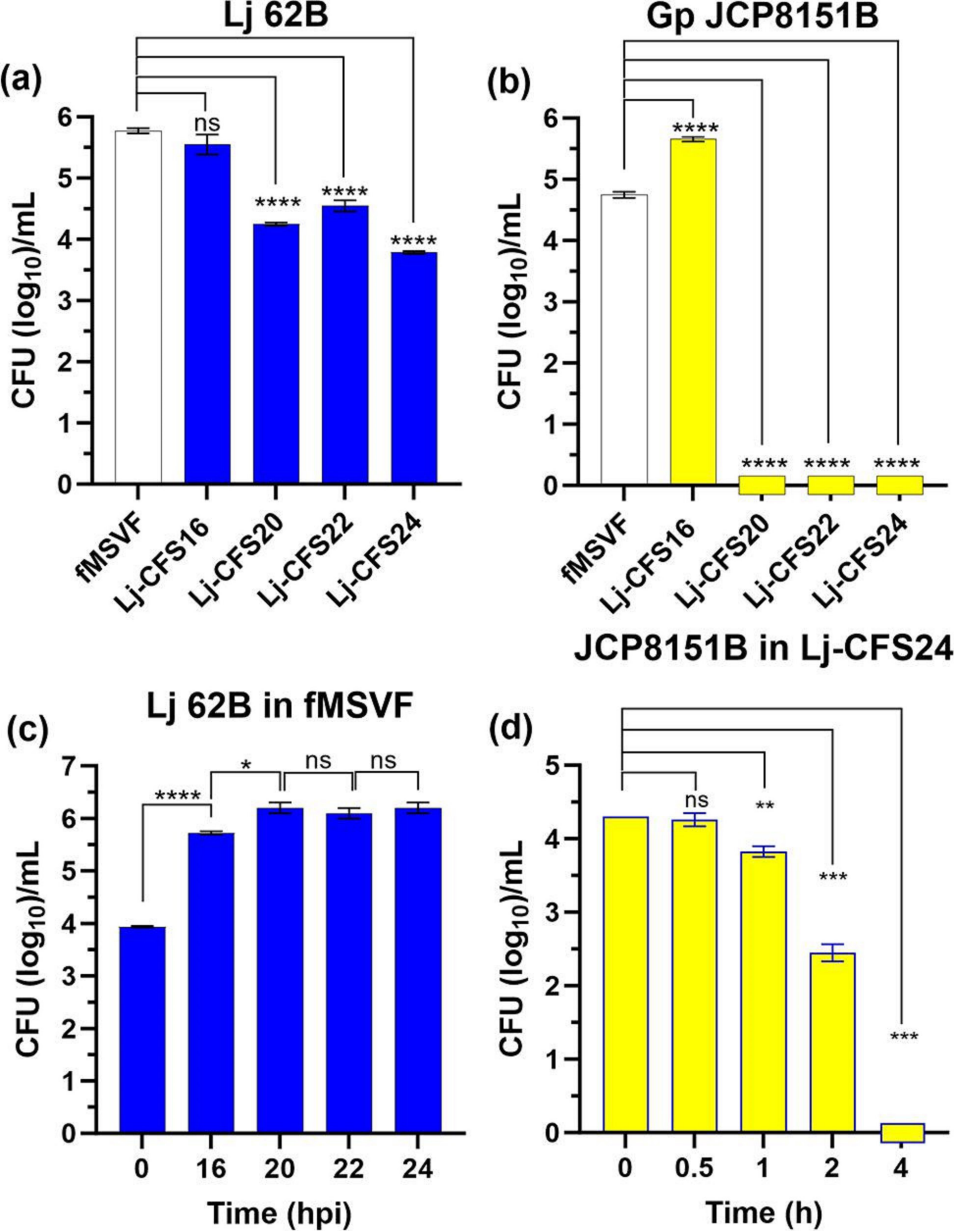
Lj-CFS obtained at 20, 22, and 24 hpi eliminated Gp JCP8151B, but not Lj 62B viability. Lj-CFS obtained at 16, 20, 22, and 24 hpi and fMSVF were inoculated with 10^4^ c.f.u. ml^−1^ of Lj 62B (**a**) or Gp JCP8151B (**b**), incubated for 24 h, and the c.f.u. per millilitre determined. (**c**) Production of the *Gardnerella* elimination factor coincides with entry of Lj 62B into the stationary phase of growth. Lj 62B was inoculated into fMSVF at 10^4^ c.f.u. ml^−1^, incubated for 24 h, and the c.f.u. per millilitre determined at the indicated times. (**d**) Lj-CFS24 significantly reduced Gp JCP8151B growth at 0.5, 1, 2, and 4 hpi. Gp JCP8151B was inoculated into Lj-CFS24 at 10^4^ ml^−1^ and c.f.u. per millilitre determined at indicated times. For all panels, values represent the means of three individual experiments±SEM. Significant differences were determined by one-way ANOVA with Dunnett’s multiple comparisons posttest using fMSVF (**a**) and (**b**) or 0 hpi growth (**d**) as the control, or Tukey’s multiple comparisons posttest (**c**); ns, no significant difference; *, *P*<0.05; **, *P*<0.01; ***, *P*<0.001; ****, *P*<0.0001.

Results of the co-culturing experiment suggested that an extracellular factor produced by Lj 62B was responsible for the elimination of Gp JCP8151B within 24 h of co-culturing. Therefore, further analysis of the potential factor involved concentrating the Lj 62B cell-free supernatant (Lj-CFS) and adding aliquots of the concentrated material to the Gp JCP8151B MSVF culture. However, unlike standard laboratory media, MSVF is complex and contains several major components found in vaginal fluid such as glycogen, mucin, and albumin [50]. Thus, rather than concentrating the supernatant, which would also concentrate components of the medium like mucin and possibly alter the function of the extracellular factor, we grew Gp JCP8151B in Lj-CFS, which should contain all or most of the product responsible for the observed elimination. The Lj-CFS from each time point (Lj-CFS16, Lj-CFS20, Lj-CFS22, and Lj-CFS24 hpi) was used as an initial growth medium for Gp JCP8151B. It is possible that the growth of Lj 62B in MSVF for up to 24 h would deplete the nutrients required for the growth of Gp JCP8151B. To exclude this possibility, we filtered MSVF (fMSVF) and used it as a growth medium control. Lj 62B and Gp JCP8151B were inoculated into fMSVF or Lj-CFS from each time point and the c.f.u. per millilitre determined 24 hpi. The growth of Lj 62B in Lj-CFS20, Lj-CFS22, and Lj-CFS24 was significantly lower than that in fMSVF (Fig. 2a). In contrast, growth of Gp JCP8151B in Lj-CFS16 was significantly enhanced compared to its growth in fMSVF (Fig. 2b). However, no c.f.u. of Gp JCP8151B were recovered 24 hpi from Lj-CFS20, Lj-CFS22, or Lj-CFS24 (Fig. 2b).

These results not only strongly support the hypothesis that upon its growth in MSVF, Lj 62B produced an extracellular factor that eliminated Gp JCP8151B but also suggest that the production of this factor occurs abruptly rather than gradually as there were only 4 h between growth enhancement by Lj-CFS16 and growth elimination by Lj-CFS20 (Fig. 2b). Production of the *Gardnerella* elimination factor appears to be related to entry of Lj 62B into the stationary phase rather than leakage of intracellular contents due to overgrowth and lysis between 16 and 20 hpi, as its growth in fMSVF reached 10^6^ c.f.u. ml^−1^ at 20 hpi and remained at that level at 22 and 24 hpi (Fig. 2c). Although the *Gardnerella* elimination factor is produced by Lj 62B at 24 hpi, it may inhibit *Gardnerella* growth if applied at earlier stages of its growth. Therefore, we inoculated Lj-CFS24 with 10^4^ c.f.u. ml^−1^ of Gp JCP8151B and determined the c.f.u. at 0.5, 1, 2, and 4 hpi. At 0.5 hpi 10^4^ c.f.u. ml^−1^ were recovered but at 1 hpi, the c.f.u. were reduced by 0.4 logs and by 1.9 logs at 2 hpi with complete eradication occurring by 4 hpi (Fig. 2d). The gradual reduction in Gp JCP8151B c.f.u. per millilitre between 0.5 and 4 h of incubation suggests that the action of the elimination factor is relatively time dependent.

**Fig. 3.**
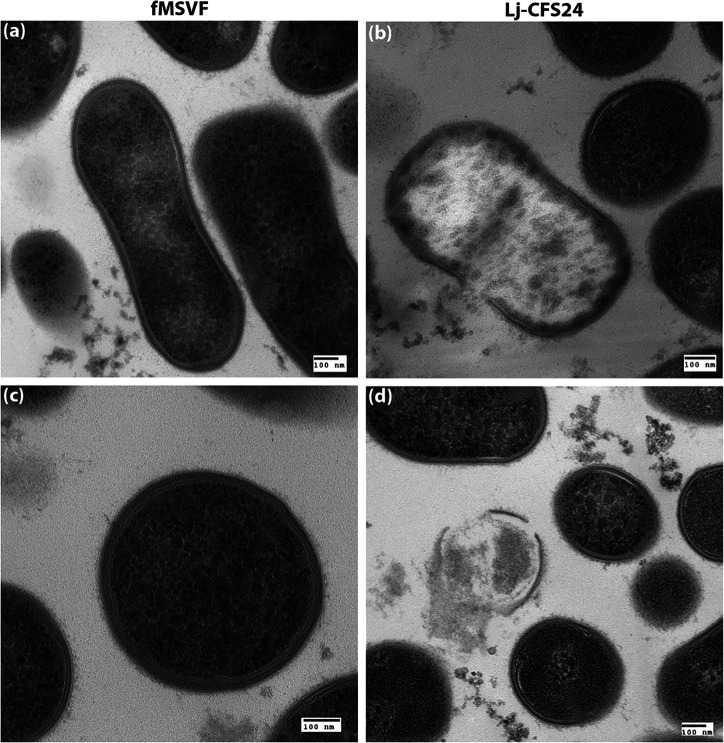
Transmission electron microscopy (TEM) shows cell lysis is the apparent cause of Gp JCP8151B elimination. Longitudinal (**a**) and (**b**) and cross-sections (**c**) and (**d**) of Gp JCP8151B grown in fMSVF (**a**) and (**c**) or Lj-CFS24 (**b**) and (**d**) are shown.

Compared to its growth in fMSVF, the growth of Gp JCP8151B in Lj-CFS24 was reduced by 1 and 2 logs at 1 and 2 hpi, respectively, and eliminated by 4 hpi (Fig. 2d). This pattern of reduction suggested that lysis of the cells may be occurring. To determine if this is the case, we performed transmission electron microscopy (TEM). While the cells from the 2 hpi time point would seem to be ideal for TEM analysis, we failed to obtain a sufficient pellet from this time point even when we pooled several batches of the culture. Therefore, we obtained the pellet from the 1 h time point. Cells from fMSVF and Lj-CFS24 inoculated with 10^5^ c.f.u. ml^−1^ Gp JCP8151B and incubated for 1 h at 37 °C under 5 % CO_2_ were collected, processed, and stained for TEM. TEM analysis showed that Gp JCP8151B cells grown in fMSVF maintained their integrity and their membranes appeared intact (Fig. 3a, c). On the other hand, many cells within cultures grown in Lj-CFS24 appeared to be disintegrating, with signs of pore formation (Fig. 3b) and disruption and sloughing of their membranes (Fig. 3d).

**Fig. 4.**
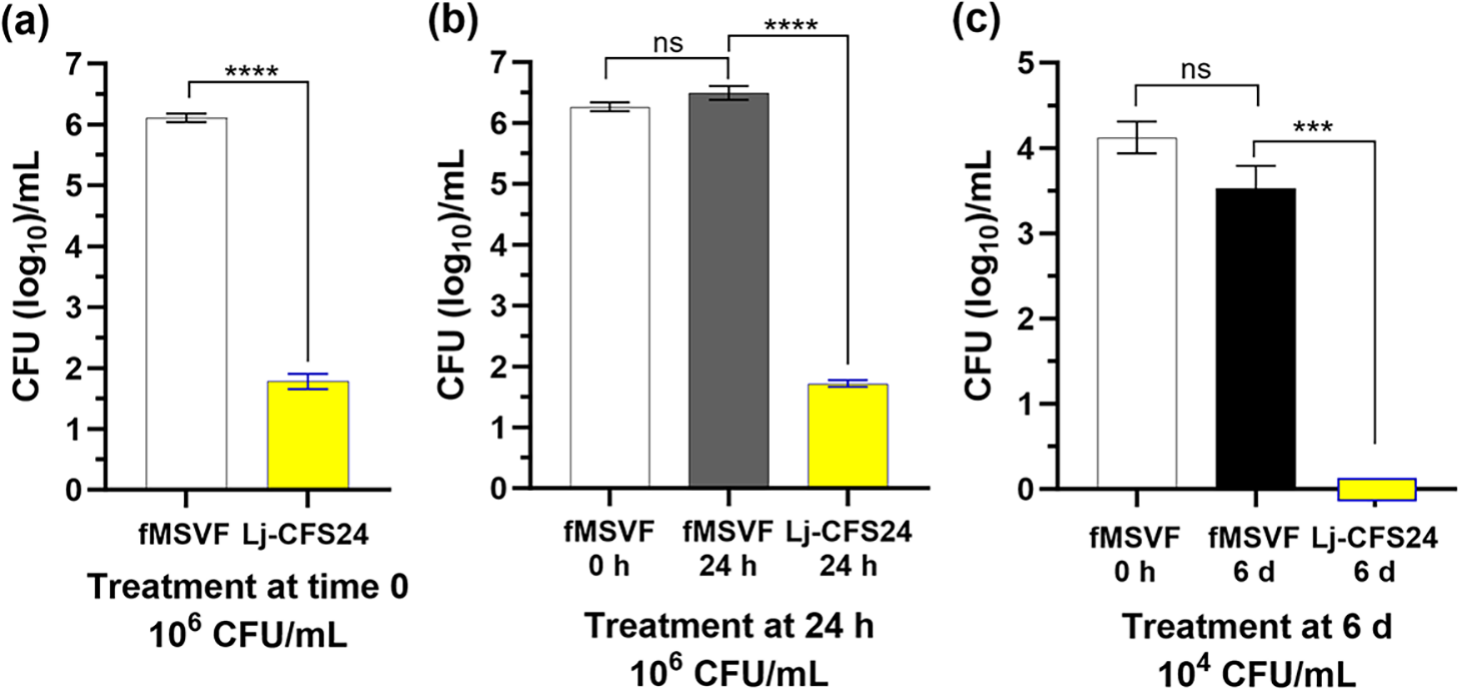
Growth in Lj-CFS24 reduces Gp JCP8151B c.f.u. whether exposed at log phase or early or late stationary phase. (**a**) Log phase: fMSVF or Lj-CFS24 inoculated with 10^6^ c.f.u. ml^−1^ incubated 24 h. (**b**) Early stationary phase: fMSVF inoculated with 10^6^ c.f.u. ml^−1^ grown for 24 h then cells harvested and resuspended in Lj-CFS24 for 24 h. (**c**) Late stationary phase: fMSVF inoculated with 10^4^ c.f.u. ml^−1^ grown for 6 d then cells harvested and resuspended in Lj-CFS24 for 24 h. Bars represent the means of three independent experiments±SEM. Significant differences between individual and co-cultures for each microorganism were determined by unpaired two-tailed *t*-test; ns, no significant difference; ***, *P*<0.001; ****, *P*<0.0001.

It is possible that the effect of the Lj-CFS24 is limited or reduced at late stages of growth of *Gardnerella* during which the growth increases and/or a potential factor(s) that may interfere with the Lj-CFS24 effect is produced. So far, we utilized an initial Gp JCP8151B inoculum of 10^4^ c.f.u. ml^−1^ in our analyses. However, through an extended 30 day growth curve analysis of Gp JCP8151B in MSVF, we demonstrated that under starting pH condition of 4.5, bacterial growth reached as high as 10^7^ c.f.u. ml^−1^ and dropped to 10^6^ over a prolonged stationary phase lasting 10 d [51]. A similar level of growth likely occurs *in vivo*. To assess the effectiveness of Lj-CFS24 under these conditions, we used an initial Gp JCP8151B inoculum of 10^6^ c.f.u. ml^−1^ (instead of 10^4^) to initiate growth in additional experiments. Compared with the untreated control culture (fMSVF), the initial inoculum of 10^6^ c.f.u. ml^−1^ was reduced by 4.3 logs at 24 hpi in Lj-CFS-24 (Fig. 4a). We then grew the initial 10^6^ c.f.u. ml^−1^ inoculum of Gp JCP8151B in fMSVF for 24 h, added 50 % (v/v) Lj-CFS24 or fresh fMSVF to the culture, and incubated them for 24 h. While the initial inoculum remained essentially the same, that of the 24 h culture of Gp JCP8151B with added Lj-CFS24 was reduced by 4.5 logs (Fig. 4b). Finally, we tested the possibility that at stationary phase of growth, Gp JCP8151B would be more resistant to the effect of the Lj-CFS24 or produce a factor(s) that interferes with its effect. We grew Gp JCP8151B in fMSVF for 6 d, added 50 % (v/v) Lj-CFS24 or fresh fMSVF, and incubated the cells for 24 h (as above). Compared with the control culture, which had not significantly changed at six dpi, we recovered no c.f.u. from the Lj-CFS24-treated culture (Fig. 4c). These results suggest that the Lj-CFS24 is effective even at increased levels of Gp JCP8151B c.f.u.

### The extracellular inhibiting factor is neither d-lactic acid nor H_2_O_2_

**Fig. 5.**
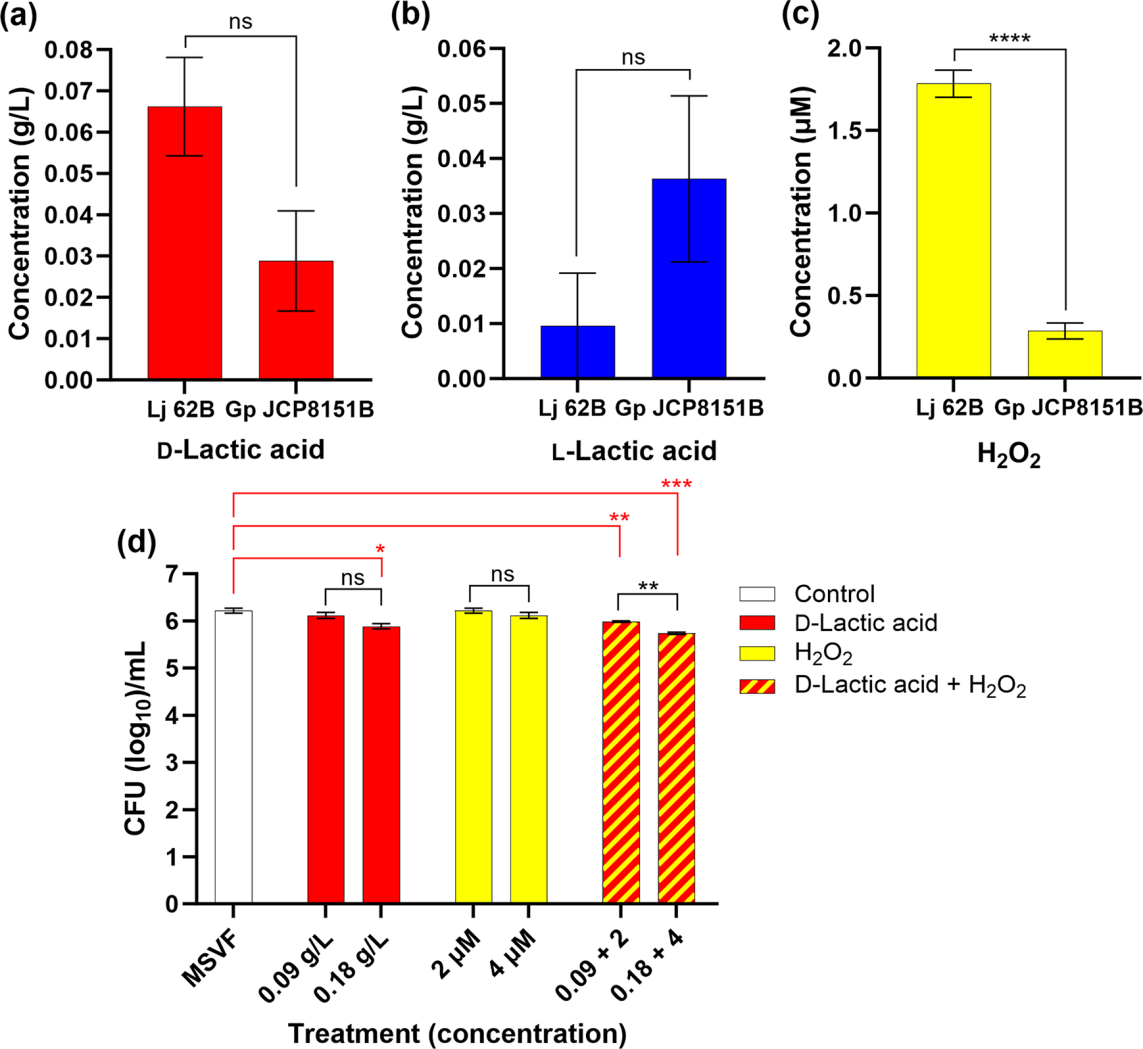
Neither d-lactic acid nor H_2_O_2_ produced by Lj 62B eliminated Gp JCP8151B growth. Strains were grown in MSVF for 24 h, the supernatants obtained, and the amount of (**a**) d-lactic acid, (**b**) l-lactic acid and (**c**) H_2_O_2_ produced by Lj 62B or Gp JCP8151B individually was determined. (**d**) d-Lactic acid (0.09 or 0.18 g l^−1^), H_2_O_2_ (2 or 4 uM), or both (0.09+2; 0.18+4) were added to the MSVF prior to inoculation Gp JCP8151B. Cultures were incubated for 24 h. For all panels, values represent the means of three individual experiments±SEM. Significant differences between pairs were calculated using two-tailed unpaired *t* tests (black); one-way ANOVA with Dunnett’s multiple comparisons posttest was used to compare each treatment to MSVF (red); ns, no significant difference; *, *P*<0.05; **, *P*<0.01; ***, *P*<0.001; ****, *P*<0.0001.

Lactic acid produced by the vaginal lactobacilli is found in both the d- and l-forms and helps maintain a low acidic environment within the vagina [7]. In addition to lactic acid, H_2_O_2_ produced by lactobacilli has been associated with lower levels of pro-inflammatory cytokines [53] and may act cooperatively with lactic acid to inhibit the growth of bacterial pathogens within the vagina [11]. We first measured the amounts of these acids and H_2_O_2_ produced in MSVF by the organisms individually. Lj 62B produced six times more d-lactic acid than l-lactic acid while Gp JCP8151B produced approximately the same amount of each form (Fig. 5a, b). However, there were no significant differences in the amounts of each form of lactic acid produced by the two strains (Fig. 5a, b). Unlike d-lactic acid production, Lj 62B produced a significantly higher level of H_2_O_2_ than Gp JCP8151B (Fig. 5c). As d-lactic acid is thought to have more of a protective role than l-lactic acid [54], we examined the effect of d-lactic acid at 1× and 2× the levels produced by Lj 62B on Gp JCP8151B growth in MSVF. Neither 0.09 nor 0.18 g l^−1^ of d-lactic acid eliminated Gp JCP8151B, although its growth was reduced by 0.18 g l^−1^ compared to growth in MSVF (Fig. 5d). When grown in MSVF containing H_2_O_2_ at either similar or higher levels than that produced by Lj 62B, there was no reduction in Gp JCP8151B c.f.u. between the two treatments (Fig. 5d). As these products occur together in vaginal fluid, we examined the combined effect of d-lactic acid and H_2_O_2_ on Gp JCP8151B growth. While a small additive effect was observed (small but increasingly significant losses in c.f.u. with lower and higher levels of d-lactic acid and H_2_O_2_ in combination compared to MSVF), combined treatment did not reduce the c.f.u. by more than 0.5 log at the higher level (Fig. 5d). Although these products may offer a minor contribution, these results rule out both d-lactic acid and H_2_O_2_ as the potential Gp JCP8151B eliminating factor.

### The extracellular factor produced by Lj 62B clearly targets *Gardnerella* **species**

**Fig. 6.**
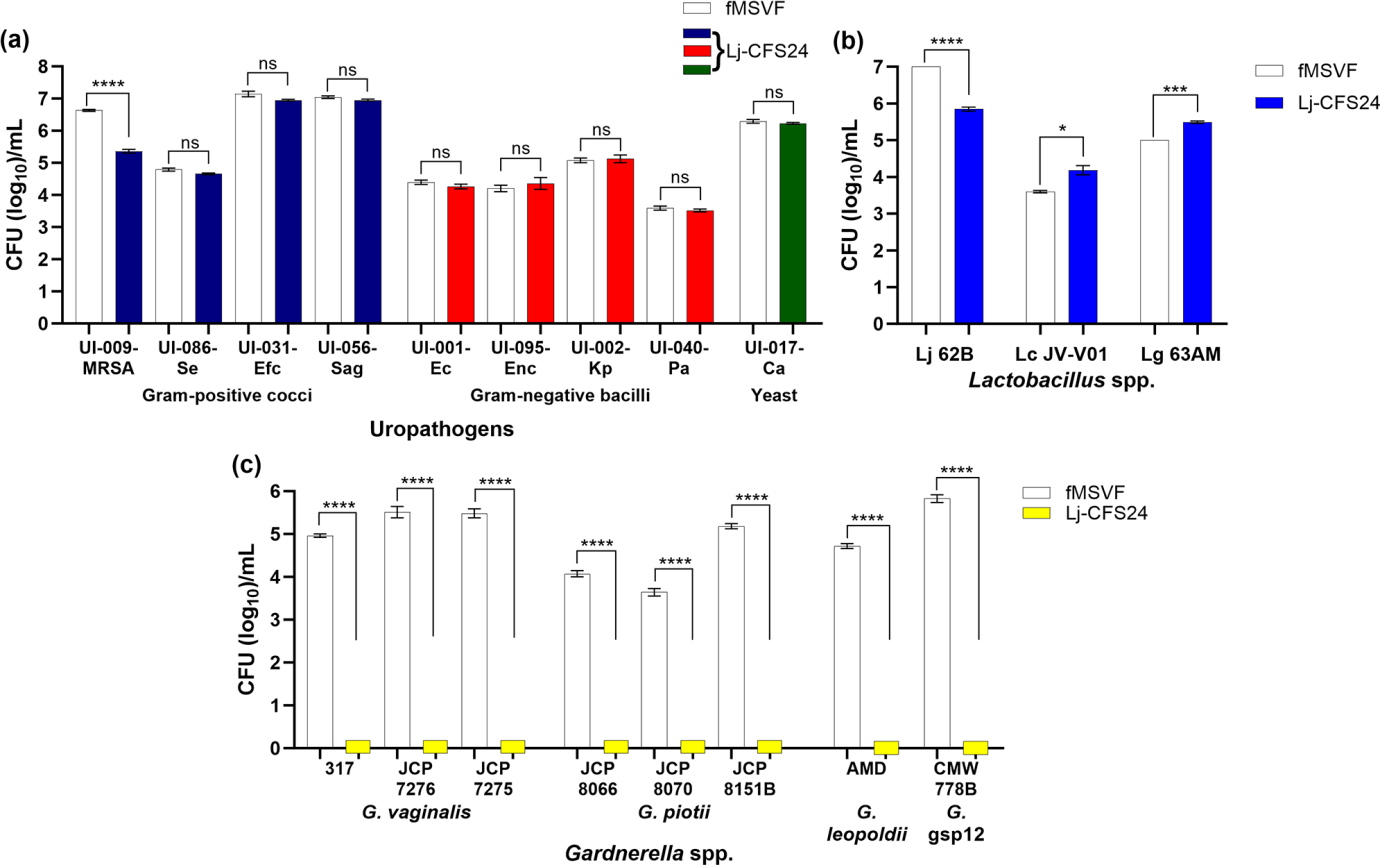
The bactericidal effect of Lj-CFS24 targets *Gardnerella* spp. (**a**) Lj-CFS24 has no broad-spectrum antimicrobial activity. Uropathogens *S. epidermidis* UI-086-Se, *E. faecalis* UI-031-Efc, *S. agalactiae* UI-056-Sag, *E. coli* UI-001-Ec, *E. cloacae* UI-095-Enc, *K. pneumoniae* UI-002-Kp, *P. aeruginosa* UI-040-Pa, and *C. albicans* UI-017-Ca were not affected by growth in Lj-CFS24 compared to their growth in fMSVF, while c.f.u. of methicillin-resistant *S. aureus* UI-009-MRSA was reduced by 1.2 logs. (**b**) The producer species and other *Lactobacillus* spp. were not eliminated by the factor in Lj-CFS24. (**c**) Lj-CFS24 eliminated the viability of all *Gardnerella* spp. strains tested; *G. vaginalis* strains 317, JCP7275, and JCP7276, *G. piotii* strains JCP8151B and JCP8070, and *G. leopoldii* AMD were obtained from women with BV; *G. piotii* JCP8066 was obtained from a healthy woman; and *Gardnerella* gsp12 from a healthy pregnant woman. For all panels, bars represent the means of three individual experiments±SEM; two-tailed unpaired *t* tests were used to determine significant differences between pairs; ns, no significant difference; *, *P*<0.5; ***, *P*<0.001; ****, *P*<0.0001.

Since neither lactic acid nor H_2_O_2_ eliminated Gp JCP8151B growth, it is possible that the extracellular factor produced by Lj 62B is a bacteriocin-like inhibitory substance (Lj-BLIS). Bacteriocins are peptides/proteins produced by different bacterial species within certain ecological niches to eliminate other competing bacterial species [14, 15, 55]. Bacteriocins produced by Gram-positive lactic acid-producing bacteria (LAB) may have a narrow or broad spectrum of activity. Some bacteriocins, like the lactococcins produced by *Lactococcus lactis *, are congener specific, acting only on other species within the genus *Lactococcus* [56–58]. Others such as thuricin from *Bacillus thuringiensis *, which targets *Clostridioides difficile *, act on one other genus [59], while bacteriocins produced by enteric LAB tend to have a broad spectrum of activity, targeting both Gram-negative and Gram-positive bacteria [17, 60]. To determine whether the host range of the Lj-BLIS is broad or narrow in spectrum, we tested it by growing nine uropathogens, three species of *Lactobacillus *, and eight strains of four *Gardnerella* spp. in fMSVF or Lj-CFS24 (Table 1). Except for the methicillin-resistant *S. aureus* strain UI-009-MRSA which was reduced by 1.2 logs, the growth of the uropathogens *S. epidermidis* UI-086-Se, *E. faecalis* UI-031-Efc, *S. agalactiae* UI-056-Sag, *E. coli* UI-001-Ec, *E. cloacae* UI-095-Enc, *K. pneumoniae* UI-002-Kp, *P. aeruginosa* UI-040-Pa, and *C. albicans* UI-017-Ca in Lj-CFS24 at 24 hpi was comparable to that in fMSVF, suggesting that the Lj-BLIS lacks broad-spectrum activity (Fig. 6a). Lj-CFS24 reduced the growth of the producer strain *L. jensenii* (Fig. 6b). However, the c.f.u. of *L. gasseri* and *L. crispatus* in Lj-CFS24 were higher than those in fMSVF (Fig. 6b). Despite these differences, Lj-CFS24 clearly sustained the growth of all three *Lactobacillus* spp. suggesting that the factor does not act on congener species. However, like its effect on Gp JCP8151B, and while the c.f.u. of the other tested *Gardnerella* strains in fMSVF varied between 3.6 (JCP8070) and 5.8 logs (CMV778B), we recovered no c.f.u. of *G. vaginalis* 317, *G. vaginalis* JCP7275, *G. vaginalis* JCP7276, *G. piotii* JCP8066, *G. piotii* JCP8070, *G. leopoldii* AMD, and *Gardnerella* gsp12 CMV778B when the strains were grown in Lj-CFS24 (Fig. 6c). We attempted to examine the effect of Lj-CFS24 on the growth of the vaginal anaerobe, *Prevotella bivia *. However, none of the three *P. bivia* strains which we purchased from the BEI, grew in fMSVF under anaerobic conditions (data not shown). Still, our collective results strongly suggest that the Lj-BLIS clearly targets *Gardnerella* spp.

### Characterization of the Lj-BLIS within Lj-CFS24

**Fig. 7.**
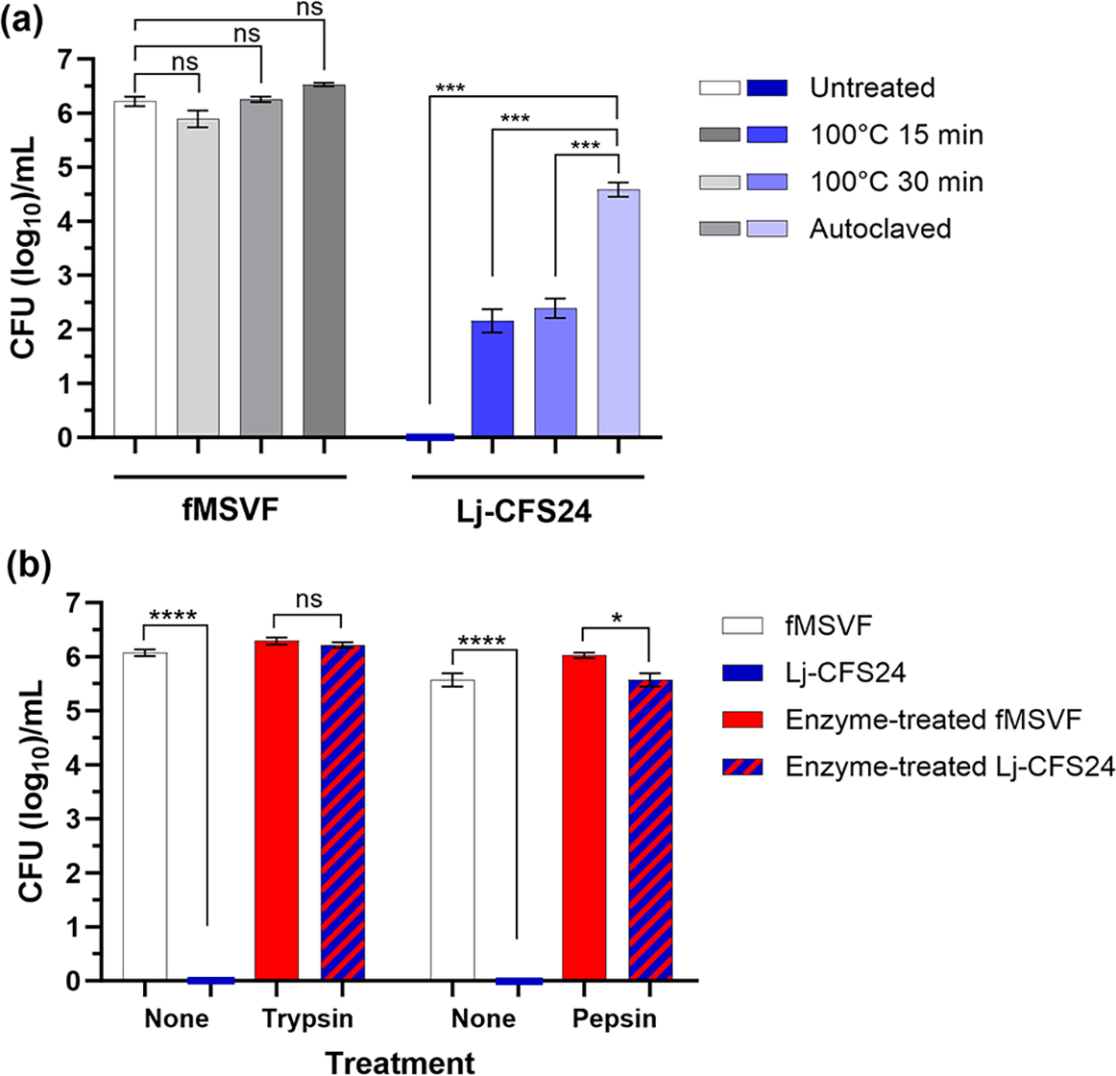
The bacteriocin-like inhibitory substance within Lj-CFS24 (Lj-BLIS) is sensitive to heat and protease treatment. Samples of fMSVF and Lj-CFS24 were treated for comparison; untreated fMSVF and Lj-CFS24 served as controls. Following treatment, all samples were mixed 50 % (v/v) with untreated fMSVF and inoculated with 10^4^ c.f.u. Gp JCP8151B. (**a**) Heat treatment, boiling Lj-CFS24 for 15 or 30 min and autoclaving at 121 °C under 15 psi for 15 min interfered with the inhibitory effect of the Lj-BLIS on Gp JCP8151B. One-way ANOVA with Dunnett’s multiple comparison posttest was used to determine significant difference between the heat treatments and controls. (**b**) Protease treatment with trypsin or pepsin eliminated the effect of the Lj-BLIS on Gp JCP8151B. Two-tailed unpaired *t* tests were used to determine significant differences between pairs of protease-treated samples. Bars represent the means of three individual experiments±SEM; ns, no significant difference; *, *P*<0.5; ***, *P*<0.001; ****, *P*<0.0001.

Bacteriocins produced by different LAB are either thermostable or thermolabile. They may also be sensitive or resistant to protease treatment. Different heat treatments and treatments with the proteases trypsin and pepsin were done to characterize the Lj-BLIS. To replace nutrients degraded by any of the treatments, each treated sample, including controls, was mixed 50 % (v/v) with untreated fMSVF. Heat treatment of fMSVF did not affect the growth of Gp JCP8151B as there were no significant differences among the treatments and the untreated control (Fig. 7a). Compared to unheated Lj-CFS24 which eliminated Gp JCP8151B*,* Lj-CFS24 heated at 100 °C for either 15 or 30 min inhibited Gp JCP8151B growth by 3.7 and 3.9 logs from controls, respectively; however, autoclaved Lj-CFS24 only inhibited its growth by 1.9 logs (Fig. 7a). Still, the reduction in the inhibitory effect of Lj-CFS24 by heat treatment supports the presence of a protein that is thermolabile to some extent. In contrast, protease treatment of Lj-CFS24 with either trypsin or pepsin eliminated the inhibitory effect on Gp JCP8151B growth – its growth was comparable to that in enzyme-treated fMSVF (Fig. 7b). These results strongly suggest that the elimination of Gp JCP8151B by Lj-CFS24 is due to a thermolabile and protease-sensitive protein, the Lj-BLIS.

**Fig. 8.**
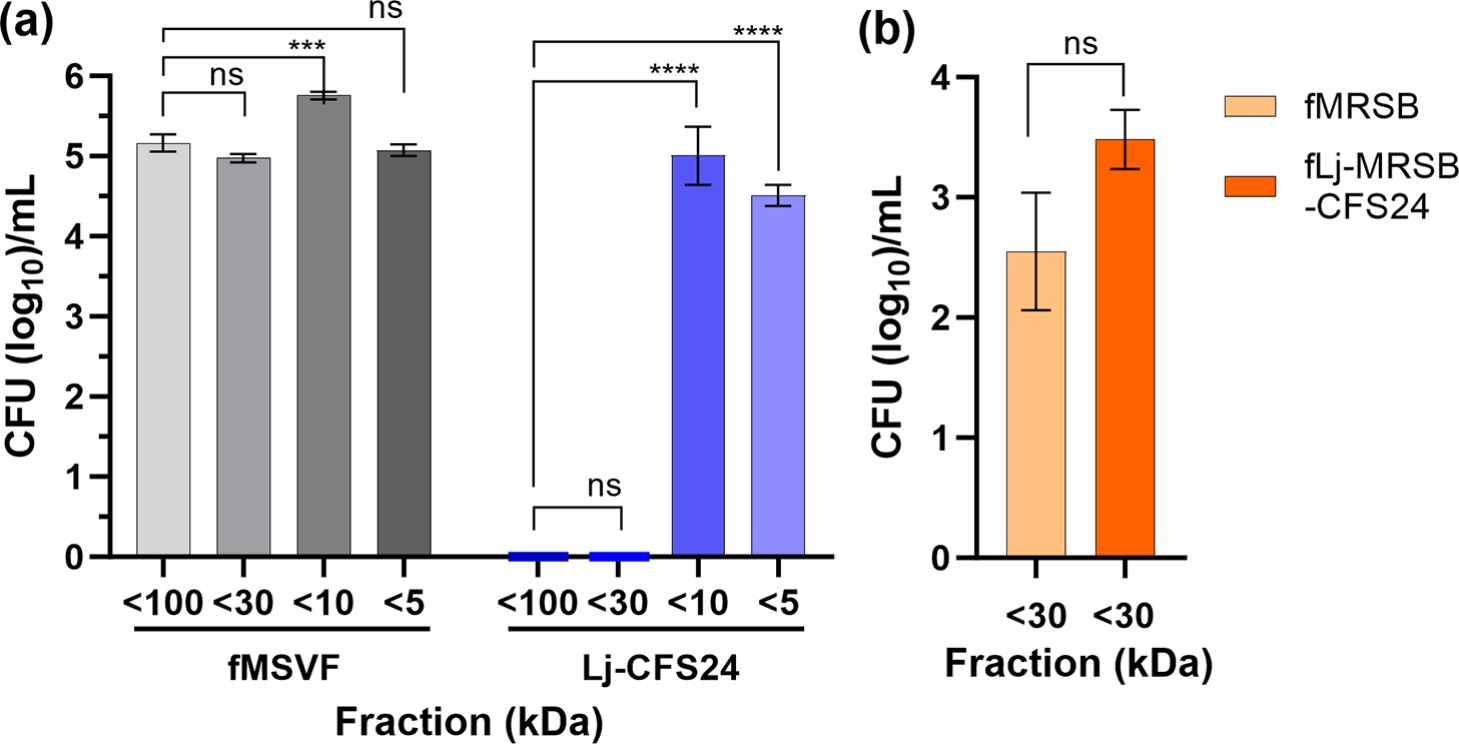
(**a**) The inhibitory effect of Lj-CFS24 on Gp JCP8151B viability resides within the 10–30 kDa protein fraction. Lj-CFS24 and fMSVF were fractionated using 100-, 30-, 10-, and 5 kDa MWCO columns. To replace essential nutrients lost during the fractionation process, each filtrate was mixed with unfractionated fMSVF 50 % (v/v). Gp JCP8151B (10^4^ c.f.u.) was inoculated in each 50 % fraction and the viability was assessed at 24 hpi. (**b**) Lj-BLIS is not produced upon the growth of Lj 62B in MRSB. Lj 62B was grown in MRSB for 24 h and cell-free supernatant was collected. This was fractionated using 30 kDa MWCO columns and the filtrate (fLj-MRSB-CFS24) was fractionated using 10 kDa cut-off columns to remove H_2_O_2_ and d-lactic acid. As a control, MRSB was processed in the same way (fMRSB). The retentates were resuspended in NCYB (6X). The fMRSB and fLj-MRSB-CFS24 were added to NYCB at 1 : 6 (v/v) in 24-well microtitre plates, inoculated with Gp JCP8151B (10^4^ c.f.u.), and viability assessed at 24 hpi. For both panels, bars represent the means of three individual experiments±SEM; one-way ANOVA with Tukey’s multiple comparisons posttest was used to compare differences among the fractions (panel a); comparison to the <100 kDa fraction are shown. Two-tailed unpaired *t*-test was used to compare the results in panel b; ns, no significant difference; ***, *P*<0.001; ****, *P*<0.0001.

Based on the results of the above experiments and considering that the Lj-BLIS is a protein or peptide, we utilized commercially available fractionation spin columns with cut off membranes of 100, 30, 10, or 5 kDa. We planned to concentrate each fraction 10× or more and add the concentrated fraction to non-fractionated fMSVF prior to Gp JCP8151B inoculation. However, despite numerous attempts, we failed to obtain sufficient amounts of the concentrated fraction to be usable. An alternative approach would be to grow Gp JCP8151B directly in each fraction. However, the fractions are not likely to support the growth of Gp JCP8151B as MSVF is a complex medium containing multiple components of variable molecular weight and the fractionation experiments would likely remove many of these components. Therefore, we used the same approach as for the heat and protease treated media; that is, adding each fraction to unfractionated fMSVF at 50 % (v/v) concentration. To ensure that this approach would not compromise interpretation of the results, we subjected fMSVF to the same fractionation procedure and added each fraction to unfractionated fMSVF at 50 % (v/v) concentration. The growth of Gp JCP8151B in each of the control mixtures was comparable, and even enhanced in the <10 kDa fraction compared to the <100 kDa fraction (Fig. 8a). In contrast, both the <100 kDa Lj-CFS24 fraction/fMSVF and the <30 kDa Lj-CFS24 fraction/fMSVF completely eliminated Gp JCP8151B c.f.u. (Fig. 8a). However, in <10 kDa Lj-CFS24/fMSVF and <5 kDa Lj-CFS24/fMSVF, the c.f.u. of Gp JCP8151B were comparable to those of their respective fractionated fMSVF/fMSVF controls (Fig. 8a). These results suggest that the BLIS produced by Lj 62B is most likely a protein within the molecular weight range of 10–30 kDa.

Lj-BLIS may also be produced by Lj 62B when it is grown under other conditions besides those mimicking the vaginal fluid (MSVF) such as in the laboratory medium MRSB. To examine this possibility, we grew Lj 62B in MRSB for 24 h, harvested the supernatant, filtered it to remove bacteria and cell debris, and fractionated it using 30 kDa MWCO columns. When grown in MRSB, Lj 62B produces sufficient H_2_O_2_ (0.03 kDa) and d-lactic acid (0.09 kDa) to inhibit *Gardnerella* spp. (Navarro *et al*., personal observation). Therefore, we then fractionated the filtrates using 10 kDa MWCO columns to remove these products. As a control, MRSB was processed in the same manner. As MRSB does not support the growth of *Gardnerella* spp. [51], we resuspended the retentates recovered from 10 kDa MWCO columns (fLj-MRSB-CFS24 and fMRSB) in NYCB and added them to additional NYCB (1 : 6 v/v) inoculated with Gp JCP8151B. As expected, the fMRSB (retentate resuspended in NCYB) did not support further growth of Gp JCP8151B; at 24 hpi, the initial inoculum was reduced by one log but not eliminated (Fig. 8b). More importantly, after 24 h of incubation, the growth of Gp JCP8151B in NYCB upon the addition of either fLj-MRSB-CFS24 or fMRSB was comparable suggesting that the Lj-BLIS *Gardnerella* elimination factor in Lj-CFS24 is specifically produced upon the growth of Lj 62B in MSVF only (Fig. 8b).

## Discussion

Our results suggest that upon its growth under conditions that mimic the vaginal fluid, Lj 62B produces an extracellular factor (Lj-BLIS) with a molecular weight range of 10–30 kDa and specific bactericidal activity against *Gardnerella* spp. The Lj-BLIS exhibited an unusually narrow spectrum of activity. As shown in Fig. 6b, the Lj-BLIS did not significantly inhibit the growth of either *L. crispatus* or *L. gasseri *. In addition, the Lj-BLIS did not significantly alter the growth of distantly related and unrelated uropathogens including *S. epidermidis *, *E. faecalis *, *S. agalactiae, E. coli*, *E. cloacae *, *K. pneumoniae *, *P. aeruginosa,* and *C. albicans* (Fig. 6a). However, it eliminated the growth of multiple species of *Gardnerella – G. vaginalis*, *G. piotii *, *G. leopoldii *, and *Gardnerella* gsp12 (Fig. 6c). Kaewsrichan *et al*. previously described high levels of a bacteriocin like component within the supernatant of the *L. jensenii* strain 5L08 and *L. crispatus* strain 6L07. The bacteriocin like compound produced by 5L08 was bactericidal for *G. vaginalis *, *E. coli *, and *C. albicans* [61]. The effect was independent of the level of H_2_O_2_ produced within the supernatant of 5L08 [61]; similar to the lack of killing of Gp JCP8151B by the levels of H_2_O_2_ produced Lj 62B that we observed (Fig. 5d). Matu *et al*. also described an ‘*in vitro* inhibitory activity’ against *G. vaginalis *, *Prevotella bivia *, and *Mobiluncus* spp. found within the supernatants from clinical isolates of *Lactobacillus* spp. [62]. The study showed that the inhibitory activity was abrogated by chemical and physical treatment and was likely a bacteriocin [62]. Using a chemically defined medium that resembled the vaginal fluid minus proteins, Aroutcheva *et al*. showed that *L. acidophilus* 160 produced a potential low molecular weight bacteriocin that inhibited the growth of all nine tested isolates of *G. vaginalis* [63]. However, the study did not examine the effect of the potential bacteriocin on lactobacilli or unrelated Gram-positive and Gram-negative bacterial pathogens [63]. Based on our findings (Fig. 6), the Lj-BLIS bactericidal effect is so far limited to *Gardnerella* spp. The failure of our *P. bivia* strains to grow in fMSVF under anaerobic conditions precluded us from testing the bactericidal effect of Lj-BLIS on this organism. If future testing reveals no bactericidal effect on other *Prevotella* spp. or on *Mobiluncus* spp., Lj-BLIS may represent a unique LAB bacteriocin that targets *Gardnerella* spp. only.

Based on their primary structure, molecular weight, genetic features, and type of post-translational modifications, bacteriocins produced by LAB are categorized into three main classes: class I, II, and III [16, 18]. Class I (< 5 kDa) and II (<10 kDa) are composed of low molecular weight heat stable peptides while class III consists of higher molecular weight (>10 kDa) thermolabile proteins that contain several domains for translocation, receptor binding, and enzymatic activity (lethality) [16, 18]. Based on its protease sensitivity, relative thermolability, and molecular weight range (Figs 7 and 8), we propose that Lj-BLIS is a class III-like bacteriocin. As shown in Fig. 8, its molecular weight range is higher than 10 kDa; within the range of 10–30 kDa. In addition, the potential Lj-BLIS is protease sensitive and relatively heat resistant (Fig. 7). Compared with the untreated Lj-CFS24, trypsin-treated Lj-CFS24 had no effect on Gp JCP8151B viability while pepsin-treated Lj-CFS24 reduced Gp JCP8151B viability by only 0.5 log (Fig. 7b). Additionally, exposing Lj-CFS24 to 100 °C or higher significantly interfered with its influence on Gp JCP8151B viability (Fig. 7a). There are two types of class III bacteriocins, group A and group B. Group A bacteriocins such as zoocin A, enterolysin A, lysostaphin, and millericin B are bacteriolysins (endopeptidases) that target different bonds within the peptidoglycan of the cell walls of Gram-positive bacteria [16, 18]. Zoocin A cleaves d-alanyl-l-alanine bonds; enterolysin A cleaves l-Ala-d-Glu bond in the stem peptide or d-Asp of interpeptide bridge, lysostaphin cleaves glycylglycine bonds, and millericin B has both d-alanyl-glycyl endopeptidase and *N-*acetylmuramyl-l-alanyl amidase activities [18, 64]. Group B bacteriocins do not cause cell lysis and their mechanisms of action vary. For example, caseicin inhibits DNA and protein synthesis, dysgalacticin inhibits sugar uptake and causes membrane leakage of small molecules, while the mechanism of action of helveticin J remains unknown [16]. Considering the rapidity of action of Lj-CFS24 – reduction of the Gp JCP8151B inoculum of 10^4^ c.f.u. by 2 logs at 2 hpi and complete eradication by 4 hpi by Lj-CFS24 (Fig. 2d), we suspect that Lj-BLIS is a class IIIa lytic bacteriocin. TEM analysis supports this possibility as both longitudinal and cross sections of GP8151B grown for 1 h in Lj-CFS24 show pore formation, membrane disruption, and cellular disintegration (Fig. 3). This rapid effect of the Lj-CFS24 differs from that of the non-lytic class III bacteriocin dysgalacticin [65]. Within 2 h of its addition to either exponential or stationary phases of *Streptococcus pyogenes,* dysgalacticin reduced the c.f.u. per millilitre by about 2 logs but did not eliminate its growth [65].

Experiments involving autoclaving (121 °C) the Lj-CFS24 for 15 min provided additional clues regarding the mechanism of Lj-BLIS function. As shown in Fig. 7a, the growth of Gp JCP8151B in the autoclaved fMSVF increased from 10^4^ CFU ml^−1^ to 10^6^ c.f.u. ml^−1^. However, in the autoclaved Lj-CFS24 and after 24 h of incubation at 37 °C under 5 % CO_2_, the Gp JCP8151B inoculum of 10^4^ c.f.u. ml^−1^ remained the same (Fig. 7a) suggesting that the Lj-BLIS inhibited further growth of the bacteria. These results hint at the possibility that the Lj-BLIS has both bacteriostatic and bactericidal activities and that autoclaving the sample eliminates the bactericidal, but not the bacteriostatic activity. Ocana *et al*. previously described a bacteriocin like substance produced by the vaginal *L. salivarius* subspecies *salivarius* CRL 1328 that was effective against *E. faecalis *, *E. faecium *, and *N. gonorrheae* [66]. Depending on the testing conditions, the bacteriocin like substance was either bactericidal or bacteriostatic. When it was added to a low inoculum of *E. faecalis* (10^3^ c.f.u. ml^−1^) a bactericidal effect was detected during 120 h of growth [66]. However, when it was added to a higher inoculum (10^7^ c.f.u. ml^−1^) a bacteriostatic effect was detected within the first 24 h of growth [66].

In this study, we analysed the production of the Lj-BLIS as well as its effect on *Gardnerella* spp. using MSVF which closely resembles vaginal fluid. Previous studies showed that the growth conditions, including growth medium, pH, and temperature, influence the production of bacteriocins by different LAB. For example, with respect to bacteriocin production, Goh and Phillip showed that among ten tested commercial media, *Weisella confusa* produced the highest level of bacteriocin when grown in MRSB [67]. Similarly, Mahrous *et al*. showed that the optimum conditions for bacteriocin production by *L. acidophilus* were in MRSB at a pH of 6.0, 34 °C, and 4 % phenyl acetamide [68]. Several studies produced LAB bacteriocins using MRSB and examined their effects on different bacterial pathogens using laboratory media designed for the optimal growth of those pathogens. For example, Dai *et al*. utilized MRSB for the production of pentocin ZFM94 from *L. pentosus* ZFM94 and Luria-Bertani broth to examine its effect on different Gram-positive and Gram-negative pathogens [69]. Gasper *et al*. utilized MRSB for the production of potential bacteriocins by the vaginal strain of *L. acidophilus* (KS400) [70]. However, to examine its effect on different bacteria, they utilized NYCB to examine its effect on *G. vaginalis* strains, brain heart infusion (BHI) broth to examine its effect on *S. aureus* and *S. agalactiae *, and tryptic soy broth to examine its effect on *P. aeruginosa* and *E. coli* [70]. Furthermore, Kaewsrichan utilized MRSB for bacteriocin production by the *L. crispatus* strain 15L08 and the *L. jensenii* strain 5608 [61]. However, they utilized BHI broth to examine the bacteriocin’s effect on *E *. c *oli* and *G. vaginalis *, and Sabouraud dextrose broth to examine its effect on *C. albicans* [61]. In this study, we utilized the following growth conditions for the Lj-BLIS production by Lj: MSVF medium, pH 5.0, 37 °C, and 5 % CO_2_. We utilized the same conditions to examine its effect on the growth of lactobacilli, Gram-positive and Gram-negative bacterial pathogens, *C. albicans*, and several *Gardnerella* spp. (Fig. 4). We also demonstrated that the Lj-BLIS was specifically produced upon growth of Lj 62B in MSVF, which mimics vaginal fluid, but not when Lj 62B was grown in MRSB, the standard laboratory growth medium for lactobacilli (Fig. 8).

At this time, we do not know the specific mechanism(s) that contributes to the production and/or activation of Lj-BLIS at 20 or 24 hpi of Lj 62B growth. However, it is clear that the potential mechanism(s) is not related to a significant enhancement in Lj 62B growth. As shown in Fig. 1d, Lj 62B viability gradually increased between 16 and 24 hpi. Our recent growth curve analysis of Lj 62B in MSVF over 30 d showed that growth was exponential at 24 to 48 hpi followed by a prolonged stationary phase during which Lj 62B maintain viability without a significant increase in growth over the remaining 28 d [51]. As seen with BLIS produced by *L. lactis* Gh 1 which reached its highest level of activity at the late-exponential phase [71], it is likely that Lj-BLIS production begins in mid-exponential phase (20–24 hpi) as the growth of Gp JCP8151B was significantly reduced at all of these time points (Fig. 1d). Among the future experiments that we plan to conduct is to determine if the Lj-BLIS production remains constant or declines during the prolonged stationary phase. It is possible that under *in vivo* conditions and during their colonization of the vaginal epithelium, Lj 62B produces the Lj-BLIS to prevent *Gardnerella* from establishing an infection, playing a role in contact inhibition and competition for binding to epithelial cells that occurs among these species. Those and other experiments designed to assess the specificity and efficacy of Lj-BLIS *in vivo* will be conducted using a relevant *in vivo* model such as the previously described murine model of BV [72].
